# A study of within-subject reliability of the brain’s default-mode network

**DOI:** 10.1007/s10334-018-00732-0

**Published:** 2019-02-07

**Authors:** Merel Charlotte Postema, Matteo De Marco, Elisa Colato, Annalena Venneri

**Affiliations:** 1 Department of Neuroscience, University of Sheffield, Royal Hallamshire Hospital, Beech Hill Road, N Floor, Room N133, Sheffield, S10 2RX UK; 2 Faculty of Earth and Life Sciences, VU University Amsterdam, Amsterdam, The Netherlands; 3 Present Address: Language and Genetics Department, Max Planck Institute for Psycholinguistics, Nijmegen, The Netherlands

**Keywords:** Brain imaging, fMRI, Hemodynamics, Reproducibility of results

## Abstract

**Objective:**

Resting-state functional magnetic resonance imaging (fMRI) is promising for Alzheimer’s disease (AD). This study aimed to examine short-term reliability of the default-mode network (DMN), one of the main haemodynamic patterns of the brain.

**Materials and methods:**

Using a 1.5 T Philips Achieva scanner, two consecutive resting-state fMRI runs were acquired on 69 healthy adults, 62 patients with mild cognitive impairment (MCI) due to AD, and 28 patients with AD dementia. The anterior and posterior DMN and, as control, the visual-processing network (VPN) were computed using two different methodologies: connectivity of predetermined seeds (theory-driven) and dual regression (data-driven). Divergence and convergence in network strength and topography were calculated with paired *t* tests, global correlation coefficients, voxel-based correlation maps, and indices of reliability.

**Results:**

No topographical differences were found in any of the networks. High correlations and reliability were found in the posterior DMN of healthy adults and MCI patients. Lower reliability was found in the anterior DMN and in the VPN, and in the posterior DMN of dementia patients.

**Discussion:**

Strength and topography of the posterior DMN appear relatively stable and reliable over a short-term period of acquisition but with some degree of variability across clinical samples.

## Introduction

Resting-state functional magnetic resonance imaging (fMRI) holds great potential for clinical application due to its high level of availability, relatively high spatial resolution (2–4 mm), and its non-invasive manner to capture brain function [1–4]. In addition, it does not require cognitive task performance and there is evidence that it can contribute significantly to high levels of individual classification accuracy in prodromal neurodegeneration due to Alzheimer’s disease (AD) [5–7].

The analytical procedure of resting-state fMRI is centred on low-frequency (< 0.1 Hz) fluctuations in the Blood Oxygen Level-Dependent (BOLD) signal [8, 9] that are dependent on the paramagnetic properties of deoxygenated haemoglobin [10]. Enhanced excitatory neuronal activity brings about an increase in oxygenated blood supply and a subsequent decrease in deoxygenated haemoglobin levels [11, 12]. This leads to decreased dephasing of excited spins and a subsequent increase in T2* decay time, reflected by an increase in BOLD-signal intensity [13].

Even during “rest” (i.e., the absence of a specific cognitive task), the brain maintains high levels of neuronal activity [14]. This is coherently organised into networks based on patterns of spontaneous synchronised activity in different brain areas [2]. This synchronous activity is referred to as functional connectivity, and studies on neurodegenerative conditions suggest that the patterns of connectivity reflect the underlying neural anatomy [15]. Various resting-state networks have been identified, including the visual-processing network (VPN), the sensorimotor network, the frontoparietal control network, the salience network, and the default-mode network (DMN) [16, 17]. The DMN is unique in showing a robust decrease in activity during cognitive task performance [14, 17], which is essential for successful engagement in overt cognitive processing, e.g., memory encoding [18]. Activation of the DMN also directly sustains cognitive processes, including autobiographical episodic memory retrieval [16, 19, 20], self-referential mental processing [16, 19], and mind wandering [21]. The topography of the DMN encompasses several brain areas and often an anterior and a posterior component are distinguished. Limbic areas are also involved in this network including the posterior cingulate cortex, retrosplenial cortex, hippocampus, and precuneus [22–24]. The anterior DMN centres in the medial prefrontal cortex and ventral anterior cingulate cortex [23, 25], while the posterior DMN centres in the inferior parietal lobule and several temporal lobe areas. Anterior and posterior DMNs also show distinct alterations in functional activity that appears to decrease with age in the posterior DMN, and both decreases and increases with age in the anterior DMN [26]. Such an increase in activity is believed to serve as a compensatory mechanism to optimise levels of cognitive performance along the axis of age-related neurological changes [27].

Interestingly, alterations in DMN activity, different from the ones observed in normal ageing [28], have been reported in several brain disorders, such as Alzheimer’s disease (AD) [15, 19]. AD is a progressive neurodegenerative disorder characterised, at the pathological level, by aberrant accumulation of amyloid-beta and tau proteins into extracellular amyloid plaques and intracellular neurofibrillary tangles, respectively [29]. Since initial amyloid deposition has been observed in several regions of the DMN, including posterior cingulate cortex, retrosplenial cortex, and lateral parietal cortex [30], and because diminished DMN functional connectivity has been observed in mild AD patients and patients at the mild cognitive impairment (MCI) prodromal stage of AD (see [16] for a review), measures of DMN functional activity with resting-state fMRI promise to become a possible biomarker of early AD [22, 31].

For resting-state fMRI to be successfully applied clinically, however, reliability of the acquired signal in the context of neurodegeneration needs to be established. Although several test–retest reliability studies of resting-state fMRI have been carried out in young healthy adults [1, 32–39], few studies have focused on healthy elderly [40] and MCI patients [41], and, to our knowledge, no such studies have been carried out in AD patients. Usually, the study of reliability of brain networks requires longitudinal designs and repeated follow-ups that are not always possible in clinical populations, especially those with a neurodegenerative condition characterised by progressive neural loss/dysfunction such as AD. The aim of this study, therefore, was to investigate the reliability of signal acquired with resting-state fMRI in the DMN in healthy elderly adults, MCI patients, and AD patients in the short term, following a procedure which exploits the processing of multiple runs acquired within the same fMRI session. All participants were scanned once, but across two different runs. Data were analysed using two different methodologies: seed-based linear functional connectivity and dual-regression analysis [42]. These are major methodologies widely implemented for the analysis of brain networks. Concurrent analytical approaches were chosen to maximise conservativeness and test the presence of both differences and similarities. Differences between Run 1 and Run 2 were tested by means of paired *t* tests run to compare the spatial map of each network calculated on each run. Run 1 is usually an acquisition during which the participants acclimatise to the scanner environment, whereas Run 2 may be associated with more fatigue, decreased wakefulness, and more movements. For this reason, the null hypothesis was accompanied by an alternative hypothesis testing whether significant differences in the DMN would exist between the two runs.

Voxel-based correlation between the two runs was instead calculated to test the similarities of the two sets of maps. In addition, analysis of network strength at the major hubs within each map was carried out from seed-based networks. For this purpose, further numerical indices of functional connectivity were extracted with a seed-based approach, using an anatomical atlas. Multiple measures of statistical correlation and consistency were calculated: Pearson’s correlation, Cronbach’s alpha, and intraclass correlation.

## Materials and methods

### Participants and data acquisition

Seventy-two healthy volunteers, 67 patients with MCI, and 31 patients with dementia of the AD type were recruited at the IRCCS Fondazione Ospedale San Camillo, Venice, Italy, as part of a wider project studying cognitive efficiency in physiological and pathological ageing (Grant no 42/RF-2010-2321718 by the Italian Ministry of Health to AV) and their data contributed to the Venice Lido Ageing Database. This study had received approval by the Institutional Review Board of the IRCCS Fondazione Ospedale San Camillo (Venice, Italy), (Protocol N. 11/09 version 2). Informed consent was obtained from all individual participants included in the study. Diagnostic status was established based on a consensus among clinicians. All participants, aged > 45 years, underwent complete neurological screening to rule out the presence of clinical exclusion criteria (extensively described in De Marco et al. [43]). To determine the extent of cognitive impairment, a comprehensive neuropsychological-test battery was used (see [5]), and scores derived from this battery were used to reach a consensus diagnosis.

T2*-weighted brain MRI sequences were acquired with a 1.5 T Philips Achieva MRI scanner. To enable assessment of the set exclusion criteria, T1-weighted, T2-weighted, and FLAIR scans were also obtained. Before scanning, participants were instructed to close their eyes and remain still for the whole duration of the session, but to remain awake. Resting-state fMRI scans were not preceded by any specific cognitive tasks.

The T2*-weighted images were acquired using the following scanning parameters: TR = 2 s, echo delay time = 50 ms, flip angle 90°, voxel dimensions 2.875 × 2.875 × 6 mm, matrix size 80 × 80 × 20, and field of view 230 mm. Two 120-volume runs of 20 contiguous axial slices obtained in ascending order were acquired for each participant. Twenty seconds of dummy scans were acquired before each run to enable longitudinal magnetisation to reach equilibrium.

A complete scanning session took approximately 35 min, during which several images were acquired. In this study, only T2*-weighted images were used for analysis. A T2*-scanning session took approximately 10 min, during which two consecutive runs were acquired, each lasting 4 min and 20 s, including 20 s during which 10 dummy volumes were acquired.

### fMRI data pre-processing

Data were pre-processed and analysed using Statistical Parametric Mapping (SPM) 8 software (Wellcome Centre for Human Neuroimaging, London, UK) implemented in MATLAB R2014a (Mathworks Inc., UK). All echo planar scans were corrected for slice timing [44], and each of the two volume runs was independently realigned [45]. This option allows the creation of mean volumes as reference and the estimation of six linear and rotational rigid body motion parameters that were visually inspected to identify problematic head movements. Participants with a translational and/or rotational movement that exceeded 1.5 mm or 3°, respectively, were excluded from the analysis. These included two healthy participants, five MCI patients, and two AD dementia patients. This is a procedure that is widely used to minimise the impact of excessive motion [46–50]. One additional patient with dementia and one additional control were excluded because of signal artefacts. The final data set included 69 healthy participants (27 males; mean age = 66 years ± 8.40; mean MMSE-score = 29.09; mean education level = 11.41 years), 62 MCI patients (30 males; mean age = 74 years ± 6.22; mean MMSE-score = 27.73; mean education level = 10.76 years) and 28 patients with AD dementia (14 males; mean age = 75 years ± 7.50; mean MMSE-score = 21.18; mean education level = 8.11 years). Between-group matching for demographic characteristics was not necessary, because no between-group comparison was carried out.

After realignment, scans were corrected for individual brain differences using spatial normalisation, which includes co-registration of the fMRI time series with the standard SPM echo planar imaging (EPI) template, and volume-based registration to normalise the EPI data to the Montreal Neurological Institute (MNI) template with known standard space [51]. Spatial normalisation was carried out for both runs separately, using the first realigned volume of the first run and the first realigned volume of the second run as source images to match the EPI template. The normalised voxel size was set at 2.00 × 2.00 × 2.00 mm. Next, a band-pass filter between 0.008 and 0.100 Hz [14] was applied using the REST toolbox (http://www.restfmri.net) in SPM 8 to remove part of non-neural sources of variability in the BOLD signal (mostly due to cardiorespiratory factors and to slow signal drift). With specific focus on cardiorespiratory rhythms, since these are pseudoperiodic, it was assumed that both cardiac and respiratory signals would be regressed out when analysed with within-subject designs in a way similar to that routinely implemented for modelling of task-based fMRI. Finally, signal-to-noise ratio was improved by spatially smoothing the fMRI images using an isotropic Gaussian kernel of 6 mm^3^ full-width at half-maximum [52].

### Cognitive and neurostructural characterisation of the cohort

Scores obtained on representative neuropsychological tests of particular relevance in ageing and neurodegeneration of the AD type were extracted and confronted between groups. As shown in Table 1, significant clinical differences were visible across the three diagnostic groups for all measures.

**Table 1:** Cognitive characteristics of the cohort. Mean (and standard deviation) is shown for each test

	Healthy adults (*n* = 69)	MCI (*n* = 62)	AD dementia patients (*n* = 28)
Mini mental state examination	29.09 (1.21)	27.73 (4.22)	21.18 (2.54)
Category fluency test	42.80 (9.42)	29.34 (9.84)	16.46 (5.84)
WAIS—Similarities	21.30 (4.74)	19.08 (4.14)	10.52 (4.28)
Prose memory test—delayed recall	13.00 (4.61)	7.27 (4.58)	1.93 (2.81)
Rey complex figure—delayed recall	16.33 (5.55)	9.68 (13.41)	2.62 (4.03)

Clinical instruments measuring general levels of cognition, episodic memory, and semantic processing are included. All group difference were significant at a Bonferroni-corrected *p* < 0.05

The three diagnostic groups were further characterised by analysing the global volumetric properties of their brains. The T1-weighted images were segmented following the standard SPM routine, and native-space maps of grey matter, white matter and cerebrospinal fluid were quantified in millilitres using the “get_totals” command line (http://www.cs.ucl.ac.uk/staff/g.ridgway/vbm/get_totals.m). Total intracranial volumes were then computed summing up the volume of the three tissue classes, and the fraction of grey matter (an index of global brain atrophy) was then obtained by fractionating grey matter volume by total intracranial volume. Three independent-sample *t* tests were then run to compare the levels of global atrophy among the three groups. These resulted to be all significant (all *p* values < 0.05), indicating that the three groups had different levels of cerebral atrophy due to neurodegenerative processes. Based on this, it was decided not to pool all participants in a single group, but apply study procedures in a separate way for each diagnostic group.

### Approaches to fMRI processing

To address the experimental question from multiple angles, the two runs were investigated in terms of divergence and convergence (i.e., testing both differences and similarities between runs). The analysis of divergence/differences was devised mainly to rule out the presence of major discrepancies between the two runs and served as a prerequisite to the main analyses.

Two complementary analytical routes were defined for the calculation of individual maps of network functional connectivity and for testing similarities and differences between runs. A seed-based methodology was implemented as a theory-driven approach, and a dual regression was implemented as a data-driven approach. The a priori choice of seeds enabled us to focus on these regions more in detail. Conversely, the data-driven definition of maps allowed us to test similarities between runs in a voxel-by-voxel way.

#### Theory-driven approach: seed-based functional connectivity networks

**Fig. 1:**
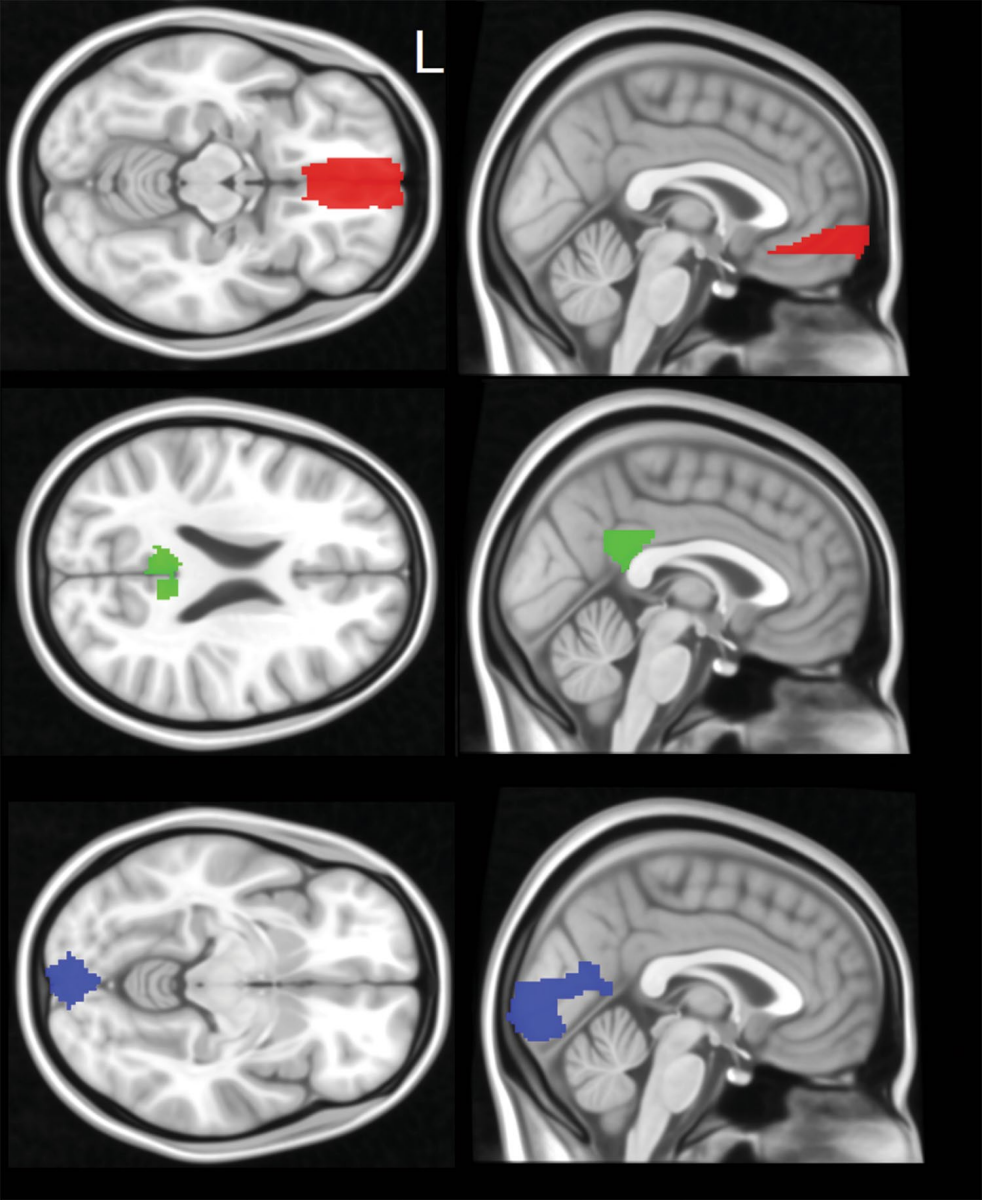
Three ROIs devised as part of the theory-driven section of the study methodology. These were constructed based on an anatomical atlas as main seed of the anterior DMN (medial prefrontal cortex, red), posterior DMN (posterior cingulate cortex, blue), and VPN (calcarine cortex, green)

A predetermined set of seed regions was selected based on the Automatic Atlas Labelling [53], and were constructed using the WFU PickAtlas toolbox [54]. The seeds included the posterior cingulate cortex and the medial prefrontal cortex for the calculation of the posterior and anterior DMN, respectively, and the calcarine cortex for the calculation of the VPN as methodological control (Fig. 1). Additional seeds were drawn in the white matter and in the cerebrospinal fluid. Seed-based timecourses were extracted from each seed region using the MarsBAR toolbox [55]. Individual maps of functional connectivity were computed modelling the linear association between the timecourse of the seed and the timecourse of each cerebral voxel. The signal extracted from the map of white matter and cerebrospinal fluid was regressed out, together with the six linear and rotational rigid body motion parameters, their squared values, their temporal difference, and the square of the differenced values, for a total of 24 regressors [56]. These regressors were calculated using the *mp_diffpow24* script (https://www.warwick.ac.uk/fac/sci/statistics/staff/academic-research/nichols/scripts/spm/mp_diffpow24.sh).

A fervent debate is still ongoing on as to whether whole-brain signal should also be regressed out from individual linear models [57]. In this study, this was not carried out, as it might lead to spurious negative correlations within individual models, which, in turn, lead to altered group-level connectivity patterns [58].

#### Data-driven approach: dual-regression networks

A second, multiple-step approach was also followed: a dual-regression procedure was carried out [42, 59] using a series of MATLAB and SPM routines. Briefly, this technique processes the spatial outline of a set of maps generated with an independent component analysis, a technique that decomposes the entire fMRI data set into a selected number of latent variables (components), each of which corresponds to an independent source of signal and has its own specific topography [6, 16]. Components of interest (corresponding to the topography of brain networks of interest) are identified, and an average individual timecourse within the contour of the component is extracted. Maps of connectivity are then calculated by modelling the linear association between this global timecourse and that of each single voxel. This was carried out regressing out the same nuisance vectors as per seed-based networks (Sect. 2.4.1). Given the data-driven nature of this approach and the profound neurological differences between healthy adults and patients, three separate independent component analyses were run, one per each diagnostic group. The fMRI toolbox GIFT (GIFT, v1.3i, http://www.mialab.mrn.org/software/gift) was used, in combination with the Infomax optimisation principle and the number of components to be extracted was set at 20, as landmark research has proficiently used this number of components to identify the major haemodynamic networks [60]. Among the 20 components, the anterior and posterior DMNs and VPN were identified by three independent raters, who obtained 100% agreement on the final selection [61].

### Inferential models

#### Investigating divergence

Paired *t* tests were carried out in SPM per group to assess individual differences in seed-based and dual-regression network topography between runs, using significance thresholds of *p* = 0.0005 (uncorrected) at the set level, and *p* = 0.05 (false discovery rate corrected) at the cluster level, as reported by the SPM output. False discovery rate was preferred over familywise error correction, because it defines a more liberal threshold for rejecting the null hypothesis [62]. As far as the specifications of this study are concerned, however, (i.e., finding confirmatory evidence in support of similarity between runs), this translated into the more conservative choice. According to the alternative hypothesis, significant differences would exist given that participants tend to experience fatigue, decreased wakefulness, and more movements during Run 2.

#### Investigating convergence

Initially, the similarity between the two maps was qualitatively inspected by running a conjunction analysis between maps generated from Run 1 and Run 2. This was done for all maps of connectivity.

Second (and in specific compliance with the remit of the hypothesis), correlational models were devised to analyse statistical similarities between Run 1 and Run 2. These were implemented as voxel-based whole-brain correlations as part of the data-driven approach (processing, therefore, maps of connectivity created after dual regression), and as regional correlations limited to the ROIs as part of the theory-driven approach (thus processing maps of connectivity obtained with seed-based models.

##### Theory-driven approach: consistency of ROIs’ statistics

Following the definition of seed-based maps, local indices of the three seeds (described in Sect. 2.4.1 and illustrated in Fig. 1) were further analysed. Data were extracted from these three ROIs for each participant individually and exported to MATLAB. IBM SPSS Statistics 23 was further used for statistical analysis. To examine if Run 1 and Run 2 were correlated with one another, a bivariate Pearson’s correlation model was initially carried out. As a short-term within-subject design was used, there was no need to correct for factors like age and AD pathology. Since Pearson’s product-moment solely examines a relationship between runs and does not indicate internal consistency, other indices of reliability were computed, namely, intraclass correlation and Cronbach’s alpha [63, 64]. Indices of reliability were run in the entire cohort and within each diagnostic group. Intraclass correlation was set to capture absolute agreement reliability in the numerical estimate between runs.

##### Data-driven approach: global and voxel-based whole-brain correlations

Both global and regional properties of individual maps of functional connectivity obtained from the dual regression were analysed. First, individual maps of connectivity were reshaped into unidimensional data matrices for the calculation of coefficients of correlation across the entire network map of each participant. These were then plotted at a group level to characterise the global strength of correlation coefficients. Second, voxel-by-voxel correlations were tested with the Biological Parameter Mapping toolbox [65], fully implemented in SPM. This toolbox allows the calculation of whole-brain maps of positive and negative correlations between two sets of inter-dependent maps. The presence of a positive coefficient of correlation between Run 1 and Run 2 was tested for each voxel. A statistical threshold of *r* > 0.6 was applied to focus exclusively on the strongest associations. The resulting maps were inspected and interpreted in the light of the expected map of the outcome (i.e., the outline of the network).

## Results

**Fig. 2:**
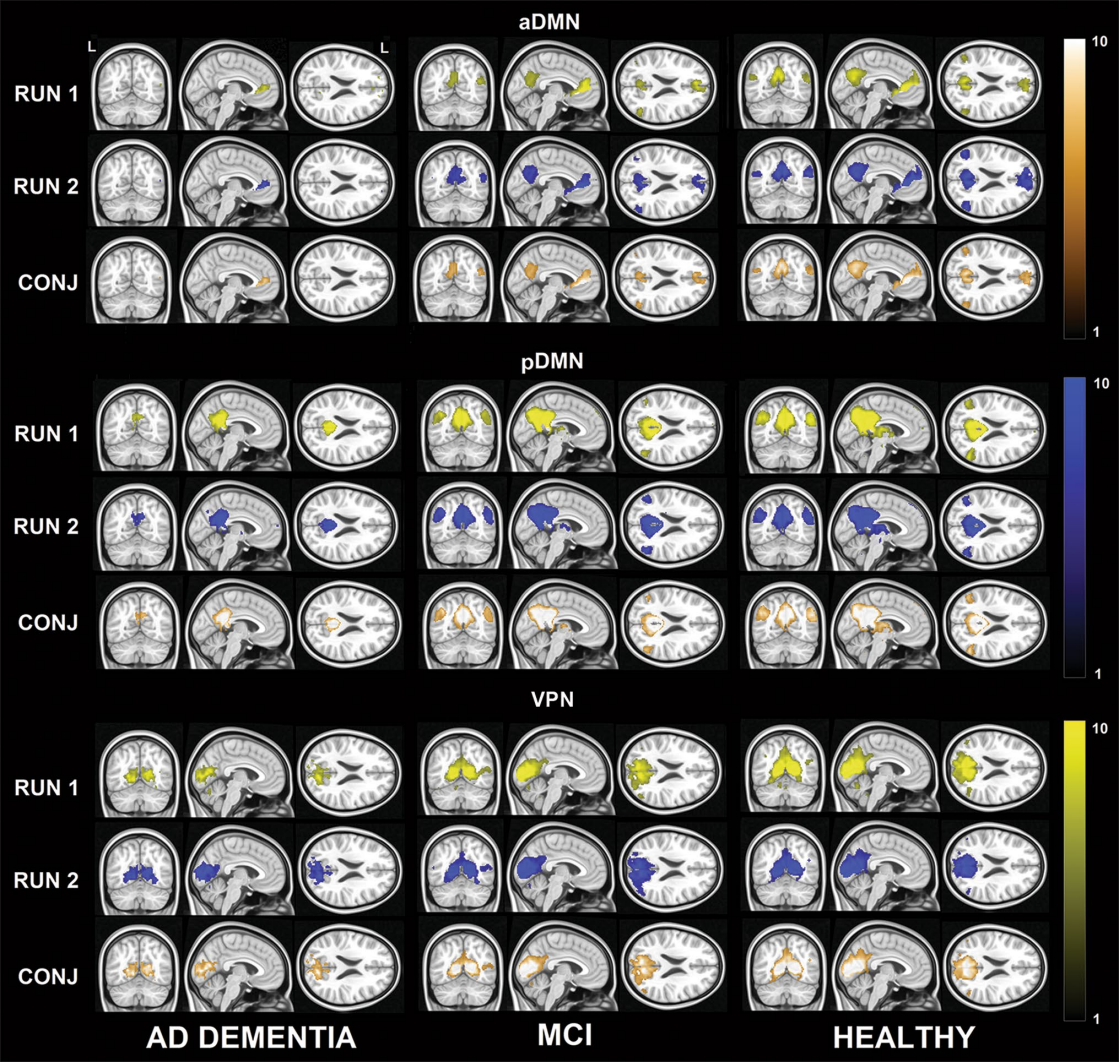
Maps of the anterior and posterior DMN and the VPN, as calculated from seed-based models via one-sample *t* tests. The strength of the haemodynamic connectivity which constitutes this network is illustrated separately for each diagnostic group: during Run 1 (yellow) and Run 2 (blue). The conjunction analysis is illustrated in gold. MNI coordinates: *x* = − 4; *y* = − 62; *z* = 22. Colours indicate the statistical strength of the *z* statistics. A legend is included on the right-hand side, *CONJ* conjunction

**Fig. 3:**
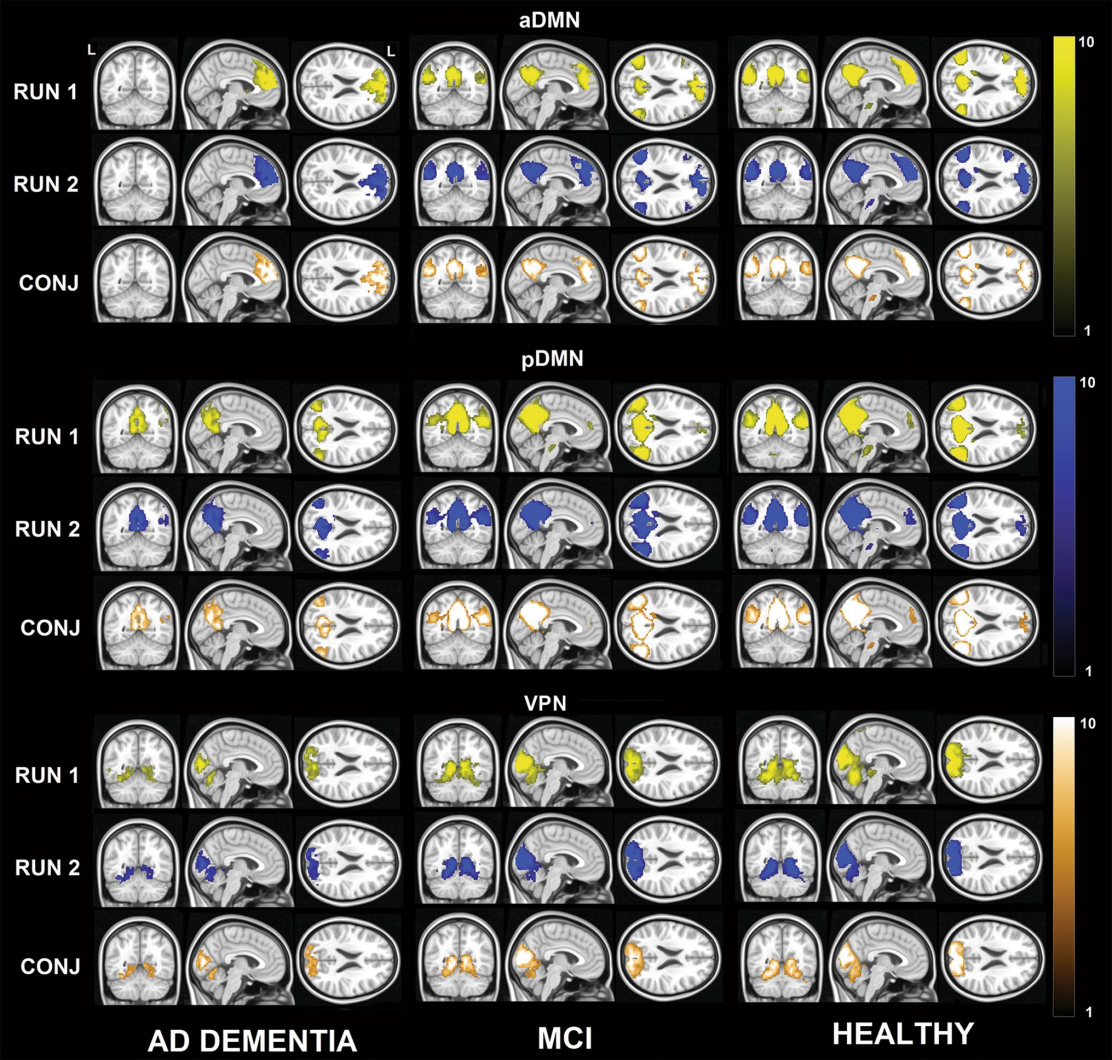
Maps of the anterior and posterior DMN and the VPN, as calculated based on the dual-regression procedure via one-sample *t* tests. The strength of the haemodynamic connectivity which constitutes this network is illustrated separately for each diagnostic group: during Run 1 (yellow) and Run 2 (blue). The conjunction analysis is illustrated in gold. MNI coordinates: *x* = *− *4; *y* = − 56; *z* = 22. Colours indicate the statistical strength of the *z* statistics. A legend is included on the right-hand side. *CONJ* conjunction

No significant differences emerged from any of the *t* test models. Moreover, all networks of healthy elderly, MCI patients, and AD patients showed high topographical similarity between runs. This is illustrated in Figs. 2 and 3, which show the results of the one-sample *t* tests and the conjunction analyses for the maps emerged from seed-based and dual-regression procedures.

Regional ROI indices of connectivity emerged from seed-based models were plotted and inspected to verify normality across the three diagnostic groups. To do so, each distribution was visually compared to that of multiple vectors randomly extracted from a normal distribution having the same mean and standard deviation as the measured data (this was carried out using *R* environment and the “rnorm” function). In no case did the distribution of peak scores suggest breach of normality.

**Fig. 4:**
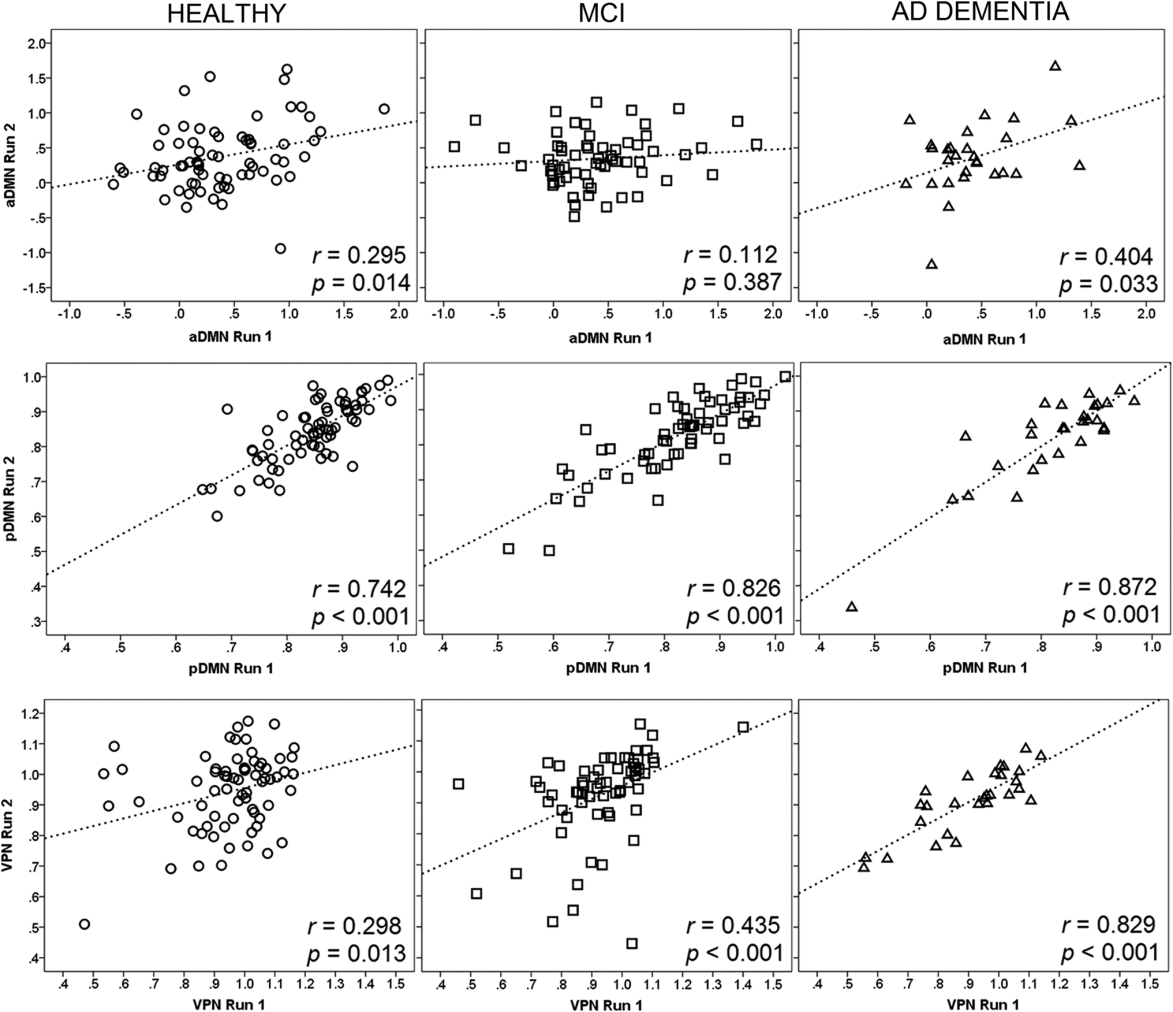
Correlation graph of mean functional activity in the central ROIs of each network (shown in Fig. 1) as emerged from seed-based models: anterior default-mode network (aDMN), posterior default-mode network (pDMN), and visual-processing network (VPN). Pearson’s correlation coefficients and corresponding *p* values are also included

Bivariate correlation analyses (illustrated in Fig. 4) revealed that the relationship between runs was very solid in the posterior DMN (*p* values < 0.001). Overall, the anterior DMN and the VPN yielded weaker coefficients of correlation. Cronbach’s alpha values and intraclass correlation coefficients are included in Table 2. Overall, the results mimicked the outcome of the coefficient models, with Cronbach’s alpha and intraclass correlation coefficient converging towards an average > 0.8 reliability for the pDMN in each diagnostic group. The reliability of both aDMN and VPN was associated with variable but overall smaller indices.

**Table 2:** Indices of reliability computed from seed-based maps of network connectivity

Reliability index	Entire cohort	Healthy	MCI	AD dementia
Anterior DMN				
Cronbach’s alpha	0.392	0.456	0.192	0.565
Intraclass correlation	0.392	0.459	0.193	0.569
Posterior DMN				
Cronbach’s alpha	0.895	0.905	0.847	0.925
Intraclass correlation	0.895	0.906	0.848	0.928
VPN				
Cronbach’s alpha	0.602	0.453	0.859	0.606
Intraclass correlation	0.604	0.456	0.863	0.610

**Fig. 5:**
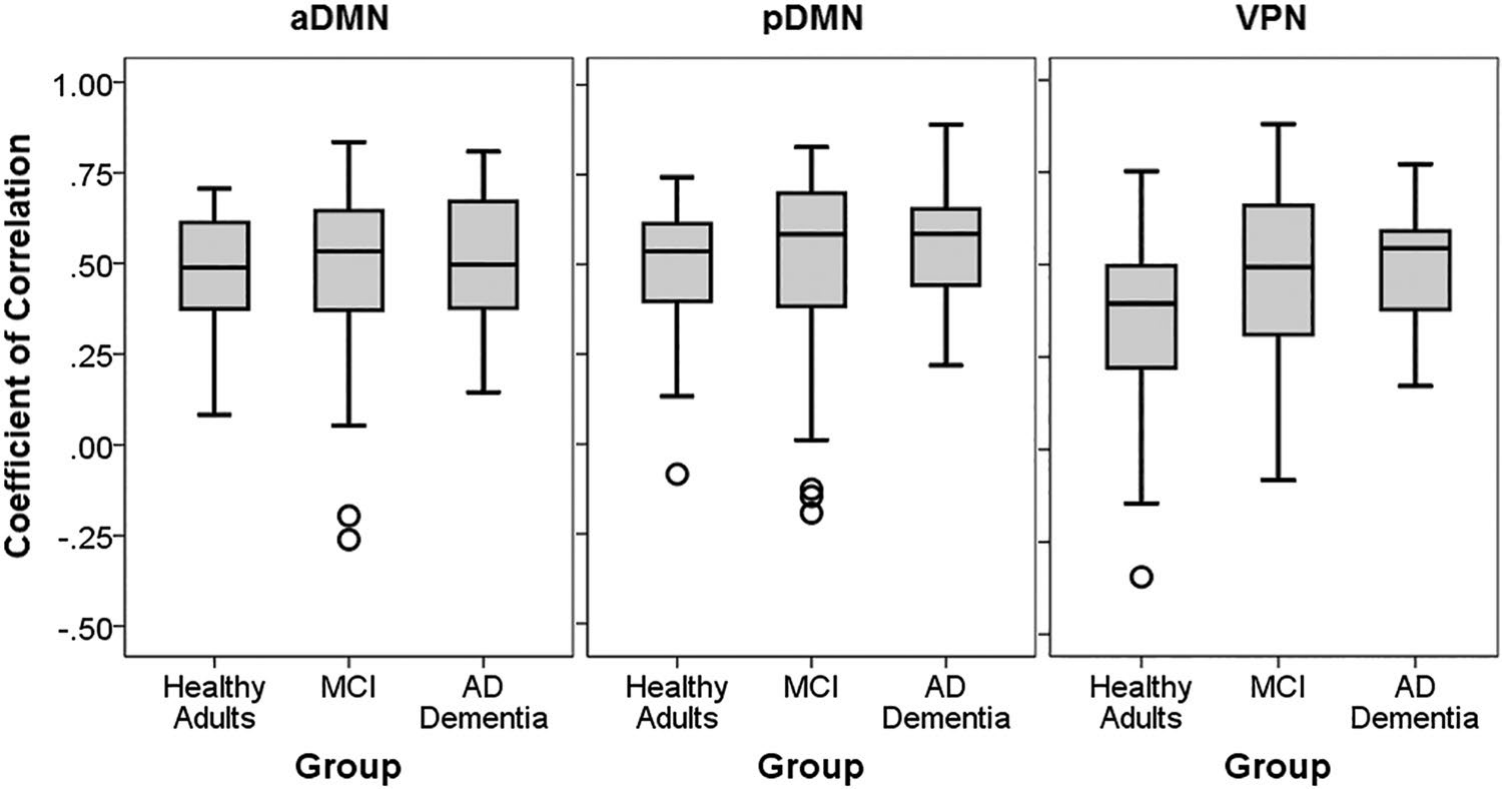
Group distribution of individual coefficients of correlation calculated over the entire network maps. Boxplots indicate medians and interquartile ranges

**Fig. 6:**
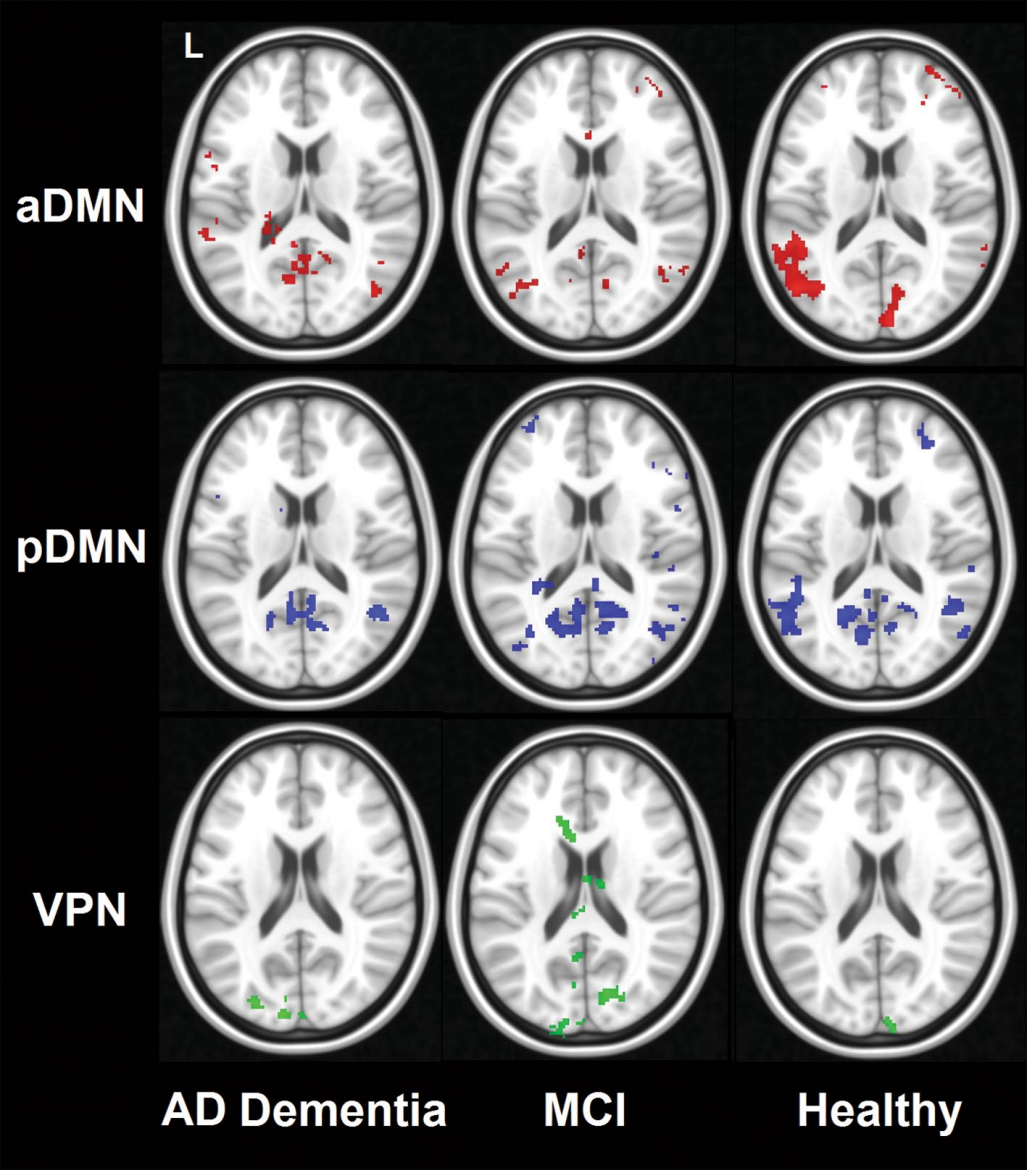
Voxel-based correlational maps between Run 1 and Run 2 for each brain network computed with dual regression. Maps were thresholded at *r* > 0.6

Individual indices of Run 1–Run 2 correlation across the entire reshaped network map are illustrated in Fig. 5 and show global robust similarity between the two runs (*r* scores tended to distribute near 0.5). The voxel-by-voxel correlation between the maps of functional connectivity calculated with dual regression on Run 1 and Run 2 is shown in Fig. 6. The results are aligned with the outcome of the ROI-based analyses: no well-defined patterns were found in association with the anterior DMN and VPN (although a trend emerged within the occipital pole for the latter network). Solid correlations within the posteromedial and inferior parietal hubs of the posterior DMN were found instead across all three diagnostic groups, interestingly with a gradual decline observed in patients with MCI and AD dementia. To test whether differences in motion between runs were the source of decreased reliability in patients, we computed for each run an index of absolute framewise displacement [66]. Briefly, this index is a measure of the average linear and rotational displacement shown over the entire run. Paired *t* test comparisons were run to compare the two runs separately for each diagnostic status. No significant differences were found.

## Discussion

In this study, we investigated short-term reliability of resting-state fMRI, with a particular focus on the DMN, in healthy elderly, MCI patient, and AD patient samples. To do so, we ran both statistical models devised to detect differences between the two runs, and statistical models devised instead to capture the degree of similarity between the two runs. In fact, the absence of significant differences between runs emerged from paired-sample *t* tests (and thus, the non-rejectability of the null hypothesis) is itself not sufficient to support the concept of analogy between runs. For this reason, paired comparisons were flanked by more conventional measures of statistical similarity. In addition, maps of network connectivity were computed using two different theory-driven and data-driven approaches: seed-based models and a dual-regression procedure, respectively.

At present, indices of functional connectivity are not recognised as validated biomarkers for AD. This study was carried out with this clinical objective in mind, in support of a potential future applicability of these measures as clinical biomarker proxies of abnormal ageing. Results indicated high within-subject reliability in the posterior DMN in healthy controls and patients, as confirmed by all statistical methods, including those testing the hypothesis of significant differences between runs, and those testing statistical similarities. The reliability of the pDMN in MCI and AD dementia, however, showed a declining trend, as confirmed in voxel-by-voxel models. Lower signal reliability was observed in the anterior DMN and in the VPN. This partially goes against our prediction of reliability measures becoming less efficient as disease progresses, because poor reliability was seen in all groups.

Since biomarker validity depends on both within- and between-subject variabilities [67], our results only partly support the applicability of the entire construct of resting-state fMRI as a potential indicator of abnormal ageing. The reason for focusing on within-subject reliability is to assess reliability while leaving out sample heterogeneity, which consequently enables generalisation of results [68, 69]. For this reason, we expected Cronbach’s alpha to be the key measure of reliability, rather than the intraclass correlation coefficient [1, 37, 40, 41]. In this study, however, the results provided by these two measures were comparable. Even though the intraclass correlation coefficient has multiple subtypes that, when chosen incorrectly, can influence reliability estimation [70], we found almost identical results across all subtypes.

Signal stability of fMRI might also be influenced by various other factors. Although AD pathology could not have influenced our results—because we used a within-subject design and scans were acquired consecutively over the span of a few minutes—factors such as machine noise, experimental instructions, data analysis strategy, and physiological noise could still have affected reliability [67]. In this study, only the latter factor could have influenced signal acquisition and could account for the observed group differences, as all participants were scanned using the same scanner, given the same instructions and analysed following the same data analysis strategy. Physiological noise encompasses cardiac-, respiratory-, and head motion and is known to increase with increasing signal-to-noise ratio [71, 72]. As we used a 1.5 T system in this study, the influence of cardiac- and respiratory noise should be relatively low, and should be minimised by the use of a within-subject design. It is likely, however, that head motion might have in part affected our data. Despite correcting for volume misalignment by means of spatial realignment, head motion is known to induce additional secondary biases, such as spin-history effects and magnetic field inhomogeneities [73]. Additional strategies have been proposed to correct for motion artefacts, aside from that implemented in this work [56, 74]. A number of these novel techniques, however, have been noted to disrupt or alter the autocorrelation structure of fMRI time series [75]. Since in-scanner motion is a major source of false positives, we are open to the possibility that the use of other methodologies may lead to slightly different findings. It is fair to acknowledge, however, that the results of the paired *t* tests were negative (i.e., no difference between runs) and that voxel-based correlation of the pDMN was limited to the network contour, and thus, false positives do not represent an issue in this study.

Signal fluctuations could have been additionally influenced by differences in eyes open and closed states, which may have resulted in significant signal differences in the VPN. Although all participants were instructed to keep their eyes closed during the whole duration of the scanning session, it is possible that some of them might have opened their eyes. McAvoy and colleagues, for instance, reported increased BOLD-signal intensity in the VPN, sensorimotor, auditory network, and retrosplenial cortex during eyes closed compared to eyes open resting states [76]. Since no camera was available in the scanner to monitor eye closure, the possible confounding influence of eye closure status on the BOLD signal cannot be ruled out. Moreover, although the definition of the DMN pattern (or that of the other networks) is easily reproducible across subjects [77], it is possible that different methodological choices (i.e., in the selection of the ICA optimisation principle or number of components, or in the use of a comparable yet different seed, e.g., the entire posterior cingulate cortex rather than the sole retrosplenial portion) may lead to slightly different results.

In addition, neuronal factors such as cognition and behaviour could have influenced within-subject signal fluctuation, as resting state is associated with unconstrained thoughts and subsequent unpredictable behaviour [78]. Nevertheless, it is unlikely that spontaneous behaviour constitutes the main source of resting-state fMRI BOLD-signal variance in this study, since robustness and spatial coherence of this type of signal have been reported in non-human primates and across various behavioural states (see [67] for a review). In addition, we have demonstrated particularly high within-subject reliability in higher order brain networks, consistent with other resting-state fMRI reliability studies (see [67] for a review).

In addition, false-positive findings and low power could have affected reliability. A recent article by Eklund and colleagues claimed that the parametric approaches used in SPM and other programmes used to analyse fMRI data, might generate up to 70% of false-positive findings, which is much larger than the generally accepted 5% [79]. Their results might have immense consequences for earlier performed fMRI-based studies, yet they strengthen the non-significant findings in our study. Furthermore, sufficient power is needed to reject the null hypothesis. Since the sample size of AD patients (*n* = 28) was more than 50% smaller than that of MCI patients (*n* = 62) and healthy elderly adults (*n* = 69), it is possible that the relatively low power in the AD group increased the likelihood of non-significant findings in this sample [80]. Zandbelt and colleagues, however, have reported that a sample size of 30 would be adequate for sufficient power in studies of this kind [69].

Of the above-mentioned factors, head motion and eyes open/closed status are the most likely factors which might have affected our data, influencing aDMN and VPN at the whole-brain level. At the single-voxel level, however, signal fluctuation appeared to be relatively lower in AD patients. We tested the possibility that this decrease might have been spuriously due to larger between-run motion differences seen in patients as opposed to controls. The analysis of between-run differences in absolute displacement was convincing evidence to rule out this possibility.

It would be interesting to investigate the mechanisms underlying low signal reliability. Since the VPN is among the latest resting-state networks affected by amyloid pathology [81], yet it shows low levels of reliability, we suggest that signal reliability is not dependent on amyloid pathology per se, but that other mechanisms, which are associated with abnormal ageing, may affect its strength. This is also supported by the robust reliability found in the posterior DMN, which includes regions that are severely affected by amyloid pathology.

Thus, further research into the molecular mechanisms underlying decreased resting-state fMRI signal reliability is needed to increase insights into abnormal ageing. To obtain a complete overview of its applicability as an indicator of abnormal ageing, studies that examine between-subject variability are needed. Furthermore, future studies could examine signal reliability using different fMRI scanners with various magnetic field strengths to increase generalisation of these results further. In addition, long-term signal reliability studies should be carried out in healthy ageing to increase insights into the effect of treatment and/or disease progression on brain functional connectivity in MCI and AD patients. To examine the value of resting-state fMRI as a biomarker proxy at the individual level, one could further study signal fluctuation in single subjects, e.g., via correlational models. Voxel-by-voxel correlational maps, however, can only be calculated at a group level, not an individual level. In fact, fMRI data modelling at the individual level results in the computation of a three-dimensional map (one sole number per voxel). In addition, individual voxel-by-voxel correlations cannot be calculated along the axis of time either, since no overt association exists between the sequence of volumes of the first acquisition and the sequence of volumes of the subsequent acquisition. Consistently with these observations, in this study, we only carried out voxelwise models testing differences and similarities between runs at a group level only.

A methodological alternative to resting state is the study of task deactivations. Since the regions that de-activate during an overt task are the same that are active at rest, reliability in the pattern of deactivation may represent a surrogate methodology for the analysis of DMN network reliability. Deactivations, however, depend on the type and computational load of the task used as part of the experimental manipulation [82], and this might lead to BOLD differences that are partly due to practice effects, or inter-individual neural reserve [83]. Furthermore, no other networks could be studied with task deactivations.

Our results support the use of haemodynamic indices, computed from resting-state fMRI, as variables of interest for a future clinical translation into a potential biomarker proxy of incipient AD at the group level and demonstrate that resting-state fMRI could even be reliably applied to AD patients in short-term studies when a voxel-by-voxel approach is adopted. This would be particularly true for the posterior DMN. Certainly, classification studies with a specific focus on cross-cohort validation would provide a definite answer as to whether a DMN index might be effectively used in clinical routine. In addition, these findings may lead to a better understanding of disease mechanisms, i.e., short-term functional activity changes in elderly and demented patients, and may form the basis for future molecular research into the degree of reliability of neuronal activity and/or cerebral blood flow in abnormal ageing.

### Conclusion

Resting-state fMRI is a reliable tool to measure functional connectivity in the posterior portion of the DMN over a short-term interval. This was supported by the converging results obtained with multiple methodologies investigating network topography and strength. High reliability of the posterior DMN in MCI and AD dementia was confirmed by group comparisons investigating topography and by correlational and consistency models investigating strength. Strength of voxel-based correlation, however, was reduced compared to healthy adults. Activity in the anterior DMN and VPN, on the other hand, showed relatively low signal stability, as indicated by poor statistical reliability. The take home message is that, despite fluctuations in signal connectivity, the posterior DMN is a reliable construct. This bodes well in the potential future translation of posterior DMN indices into potential clinical biomarker proxies.
